# Genes Responsive to Low-Intensity Pulsed Ultrasound in MC3T3-E1 Preosteoblast Cells

**DOI:** 10.3390/ijms141122721

**Published:** 2013-11-18

**Authors:** Yoshiaki Tabuchi, Yuuki Sugahara, Mika Ikegame, Nobuo Suzuki, Kei-ichiro Kitamura, Takashi Kondo

**Affiliations:** 1 Division of Molecular Genetics Research, Life Science Research Center, University of Toyama, 2630 Sugitani, Toyama 930-0194, Japan; E-Mail: yuukisugaharahcv@gmail.com; 2 Department of Oral Morphology, Graduate School of Medicine, Dentistry and Pharmaceutical Sciences, Okayama University, 2-5-1 Shikata-cho, Kita-ku, Okayama 700-8525, Japan; E-Mail: ikegame@md.okayama-u.ac.jp; 3 Noto Marine Laboratory, Institute of Nature and Environmental Technology, Kanazawa University, Ogi, Noto-cho, Ishikawa 927-0553, Japan; E-Mail: nobuos@staff.kanazawa-u.ac.jp; 4 Institute of Health Sciences, College of Medical, Pharmaceutical and Health Sciences, Kanazawa University, 5-11-80, Kodatsuno, Kanazawa 920-0942, Japan; E-Mail: kkitamur@mhs.mp.kanazawa-u.ac.jp; 5 Department of Radiological Sciences, Graduate School of Medicine and Pharmaceutical Sciences, University of Toyama, 2630 Sugitani, Toyama 930-0194, Japan; E-Mail: kondot@med.u-toyama.ac.jp

**Keywords:** low-intensity pulsed ultrasound, osteoblast, microarray, gene expression, gene network

## Abstract

Although low-intensity pulsed ultrasound (LIPUS) has been shown to enhance bone fracture healing, the underlying mechanism of LIPUS remains to be fully elucidated. Here, to better understand the molecular mechanism underlying cellular responses to LIPUS, we investigated gene expression profiles in mouse MC3T3-E1 preosteoblast cells exposed to LIPUS using high-density oligonucleotide microarrays and computational gene expression analysis tools. Although treatment of the cells with a single 20-min LIPUS (1.5 MHz, 30 mW/cm^2^) did not affect the cell growth or alkaline phosphatase activity, the treatment significantly increased the mRNA level of *Bglap*. Microarray analysis demonstrated that 38 genes were upregulated and 37 genes were downregulated by 1.5-fold or more in the cells at 24-h post-treatment. Ingenuity pathway analysis demonstrated that the gene network U (up) contained many upregulated genes that were mainly associated with bone morphology in the category of biological functions of skeletal and muscular system development and function. Moreover, the biological function of the gene network D (down), which contained downregulated genes, was associated with gene expression, the cell cycle and connective tissue development and function. These results should help to further clarify the molecular basis of the mechanisms of the LIPUS response in osteoblast cells.

## 1. Introduction

In many medical fields, ultrasound (US) has proven to be an effective diagnostic or therapeutic tool [1,2]. With respect to the therapeutic applications, low-intensity pulsed US (LIPUS) has been used extensively as an adjuvant to fracture healing [3–6]. It has been well-established that fracture healing involves four events: inflammation, soft callus formation, hard callus formation and bone remodeling. Pre-clinical work has shown LIPUS to be effective in bone fracture models of small [7–18] and large animals [19–21]. Duatre [7] previously demonstrated that LIPUS accelerated bone fracture union in a rabbit femoral fracture model. Azuma and coworkers [9] also clearly demonstrated that LIPUS acted effectively at each phase of the healing process in a rat femoral fracture model. The results of a study using a sheep osteotomy model similarly demonstrated that LIPUS can significantly accelerate the fracture-healing process, increase the cortical bone mineral density and improve the lateral bending strength of the healing fracture [19]. These therapeutic effects of LIPUS have since been confirmed in randomized clinical trials [22,23].

The use of *in vitro* cell culture systems has been of central importance for research at the cellular and molecular levels. A number of investigations have indicated that LIPUS is effective for the treatment of different cell types that play important roles in the processes of fracture healing. Most *in vitro* studies have examined the US-associated bone formative response by use of osteoblastic cells from mammals [24–30]. We recently demonstrated that the osteoblast activity in goldfish scales responds sensitively to LIPUS and may be important in promoting bone formation [31]. LIPUS has been proven effective in bone marrow stromal cells, which are thought to be one of the cell types involved in fracture healing [32,33]. In addition, LIPUS is reported to stimulate the growth and synthesis of matrix proteins in chondrocytes [34,35]. LIPUS was demonstrated to exert its effects through integrin receptors; that is, LIPUS promotes cell proliferation via the activation of integrin receptors in human skin fibroblasts [36]; and LIPUS activates α_5_β_1_ integrin and promotes cell differentiation in mouse mandibular osteoblasts [30]. Moreover, LIPUS induces RANKL and chemokines via the angiotensin II type 1 receptor in MC3T3-E1 cells [25]. LIPUS has been shown to stimulate cell proliferation, proteoglycan synthesis and expression of growth factor-related genes in human nucleus pulposus HNPSV-1 cells [37]. LIPUS is reported to regulate the proliferation and differentiation of osteoblasts through osteocytes [38]. As described above, the effects of LIPUS are evident; however, the detailed mechanisms by which LIPUS promotes bone fracture healing at the cellular or molecular level are largely unclear.

With the help of novel transcript profiling technology, a view of the genome-wide expression profiles can be assayed simultaneously, allowing scanning differential expression of a large number of genes. This technology has been used to analyze the expression of genes in response to LIPUS in human osteoblastic osteosarcoma MG-63 [28] and SaOS-2 cells [29]. While it is important to identify individual genes that are differentially expressed, there is also a need to move beyond this level of analysis. Recently, we have used pathway analysis technologies to map gene expression data into relevant gene networks on the basis of their functional annotation and known molecular interactions [39–41]. Using these technologies, unique gene networks that are associated with cellular development and cell death were identified in human lymphoma U937 cells treated with LIPUS [39].

In the present study, we investigated the changes in gene expression in MC3T3-E1 preosteoblast cells treated with LIPUS by using a GeneChip^®^ microarray analysis system in order to better understand the molecular mechanisms underlying cellular responses to this stress.

## 2. Results

### 2.1. Effects of LIPUS on the Cell Growth and Alkaline Phosphatase (ALP) Activity in MC3T3-E1 Cells

**Figure 1:**
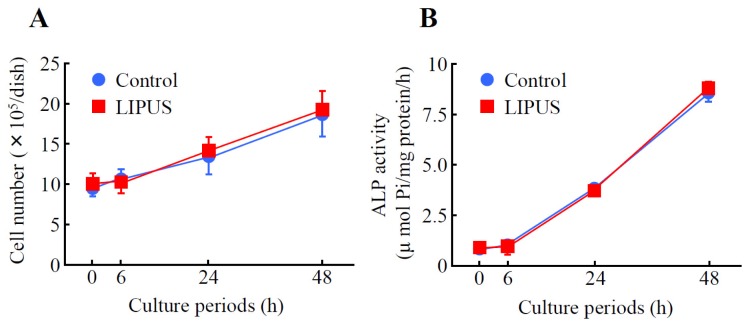
The effects of low-intensity pulsed ultrasound (LIPUS) on cell growth and Alkaline Phosphatase (ALP) activity in preosteoblast MC3T3-E1 cells. Cells were exposed to LIPUS at 30 mW/cm^2^ for 20 min followed by 0, 6, 24, and 48 h culture at 37 °C. The cell number (**A**) and ALP activity (**B**) were measured. Mock-treated cells served as a control. Data indicate the means ± SD for four different experiments.

Mouse preosteoblast MC3T3-E1 cells were analyzed to determine cell growth and Alkaline Phosphatase (ALP) activity. When MC3T3-E1 cells were exposed to LIPUS (30 mW/cm^2^, for 20 min), followed by culturing at 37 °C for 0 to 48 h, the cell number was gradually increased in a time-dependent fashion. However, the growth rate was comparable to that of the mock-treated control cells (Figure 1A). Furthermore, we examined the effects of LIPUS on ALP activity, an *in vitro* osteoblastic differentiation marker. As shown in Figure 1B, the values of control ALP activity at 0, 6, 24 and 48 h of culture were 0.80 ± 0.10, 1.1 ± 0.07, 3.8 ± 0.15 and 8.8 ± 0.33 μmol Pi/mg protein/h (mean ± SD), respectively. On the other hand, treatment of cells with LIPUS did not affect the ALP activity.

### 2.2. Effects of LIPUS on the Expression Level of mRNAs for Osteoblast Differentiation Marker Proteins

**Figure 2:**
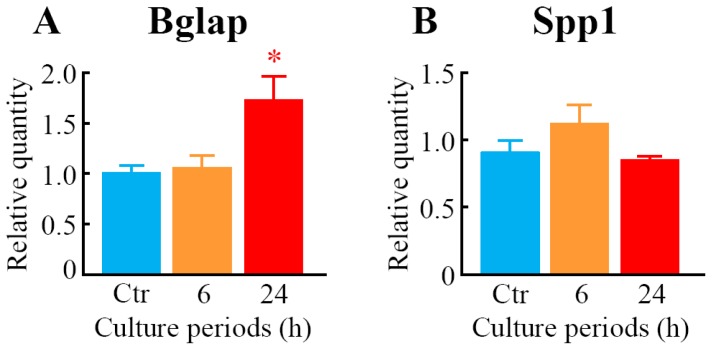
The effects of LIPUS on expression levels of mRNAs for osteoblast differentiation marker proteins. Cells were exposed to LIPUS at 30 mW/cm^2^ for 20 min followed by 6- and 24-h culture at 37 °C. Real-time quantitative PCR was carried out with specific primers for *Bglap* (**A**) and *Spp1* (**B**). Mock-treated cells served as a control (Ctr). Data indicate the means ± SD for four different experiments. * *p* < 0.05 *vs.* Ctr by the Student’s *t*-test.

We measured the expression level of mRNAs for osteoblast differentiation marker proteins, bone gamma carboxyglutamate protein (*Bglap*; osteocalcin) and secreted phosphoprotein 1 (*Spp1*; osteopontin) in MC3T3-E1 cells. After 24 h of LIPUS treatment, the mRNA level of *Bglap* was significantly elevated compared to the control (Figure 2A). However, the expression of *Spp1* was not changed by the LIPUS exposure (Figure 2B).

### 2.3. Genes Responsive to LIPUS

We carried out a global-scale oligonucleotide microarray analysis of the cells exposed to LIPUS followed by 6- and 24-h culture at 37 °C by means of a GeneChip^®^ system with a Mouse Genome 430 2.0 array, which was spotted with 45,101 probe sets. We detected 17,945, 17,970 and 17,836 (mean, *N* = 3) probe sets that were expressed in the cells treated with mock (control), LIPUS plus 6-h culture and LIPUS plus 24-h culture, respectively. These results indicated that there was essentially no difference in the numbers of expressed probe sets among the three samples. The complete lists of probe sets from MC3T3-E1 cell samples are available on Gene Expression Omnibus (GEO), a public database (accession numbers: GSE45487).

**Figure 3:**
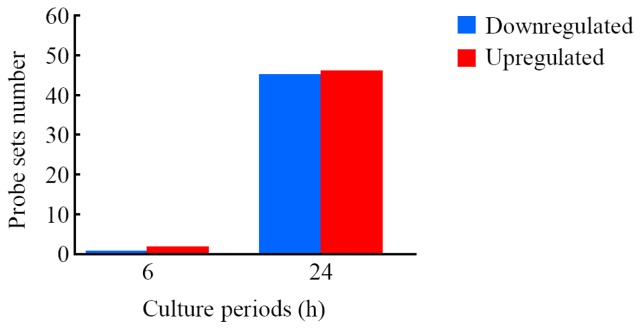
Number of probe sets that were differentially expressed in cells treated with LIPUS. Cells were exposed to LIPUS (30 mW/cm^2^, for 20 min), followed by culturing at 37 °C for six- and 24-h. Gene expression analysis of the probe sets that were upregulated and downregulated by a factor of 1.5 or greater was conducted using GeneSpring^®^ software. The number of commonly expressed probe sets affected by LIPUS from three different experiments is shown.

The expression analysis in LIPUS-treated cells using GeneSpring^®^ software demonstrated many probe sets that were differentially expressed by a factor of 1.5 or greater. The Venn diagrams in Figure S1 summarize the numbers of probe sets that were upregulated (Figure S1A,C) and downregulated (Figure S1B,D) in response to LIPUS treatment (6- and 24-h culture) in all three experiments. After 6-h LIPUS treatment, only one (ciliary rootlet coiled-coil, rootletin (*Crocc*)) and two probe sets (gelsolin (*Gsn*) and IQ motif and Sec7 domain 2 (*Iqsec2*)) were found to be upregulated and downregulated, respectively. On the other hand, 45 and 46 probe sets were found to be upregulated and downregulated, respectively, in the cells treated with LIPUS followed by 24-h culture. Lists of the probe sets that were upregulated and downregulated are shown in Tables 1 and 2, respectively. From these data, we found that one and 38 genes were upregulated or two and 37 genes downregulated after 6- and 24-h LIPUS exposure, respectively (Figure 3).

### 2.4. Identification of Biological Functions and Gene Networks

Functional category analysis of upregulated and downregulated genes in response to LIPUS followed by 24-h culture at 37 ° was conducted by using the Ingenuity^®^ Pathways Knowledge Base. The numbers of functionally annotated genes among the 38 upregulated and 37 downregulated genes were 29 and 27, respectively (Tables S1 and S2). Five biological functions, *i.e.*, skeletal and muscular system development and function (number of genes: 16), cellular movement (17), connective tissue development and function (12), embryonic development (14) and organ development (13) were observed in the functionally annotated upregulated genes (Table S1). In the functionally annotated downregulated genes, the biological functions were determined to be gene expression (number of genes: four), cell cycle (10), connective tissue development and function (7), cellular development (16) and cellular growth and proliferation (18) (Table S2).

**Figure 4:**
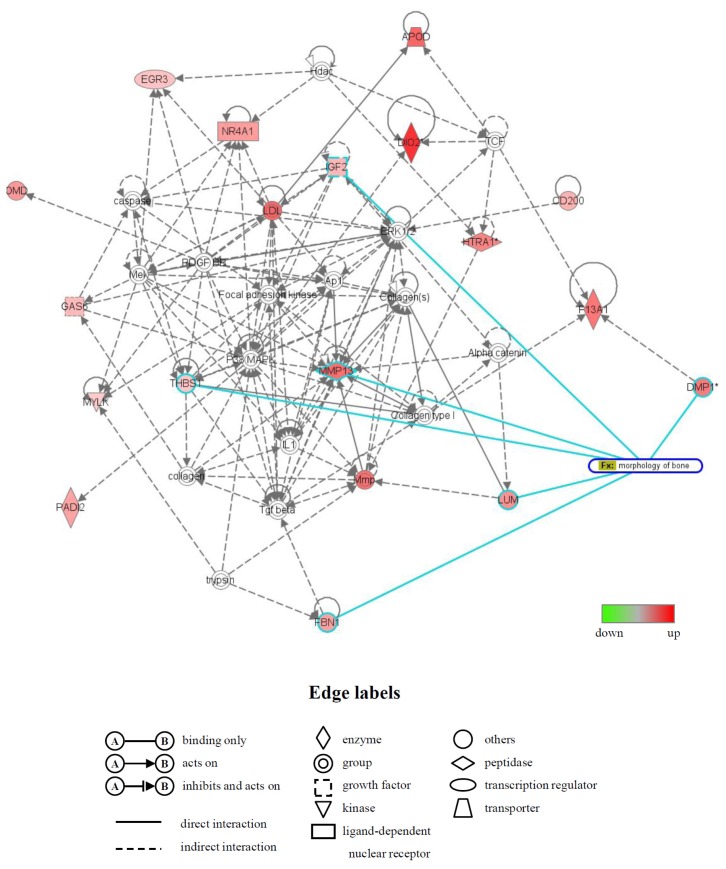
A network of genes that were upregulated in the LIPUS-treated cells. Upregulated genes were analyzed by Ingenuity^®^ Pathways Analysis tools. The gene network UP is shown. In the graphical representation of the networks, nodes refer to genes and edges refer to the biological relationships between nodes. The node color demonstrates the expression level of genes. Nodes and edges are displayed in various shapes and labels reflecting the functional class of each gene and the nature of the relationships involved, respectively. Genes that were mainly associated with bone morphology are highlighted in blue.

**Figure 5:**
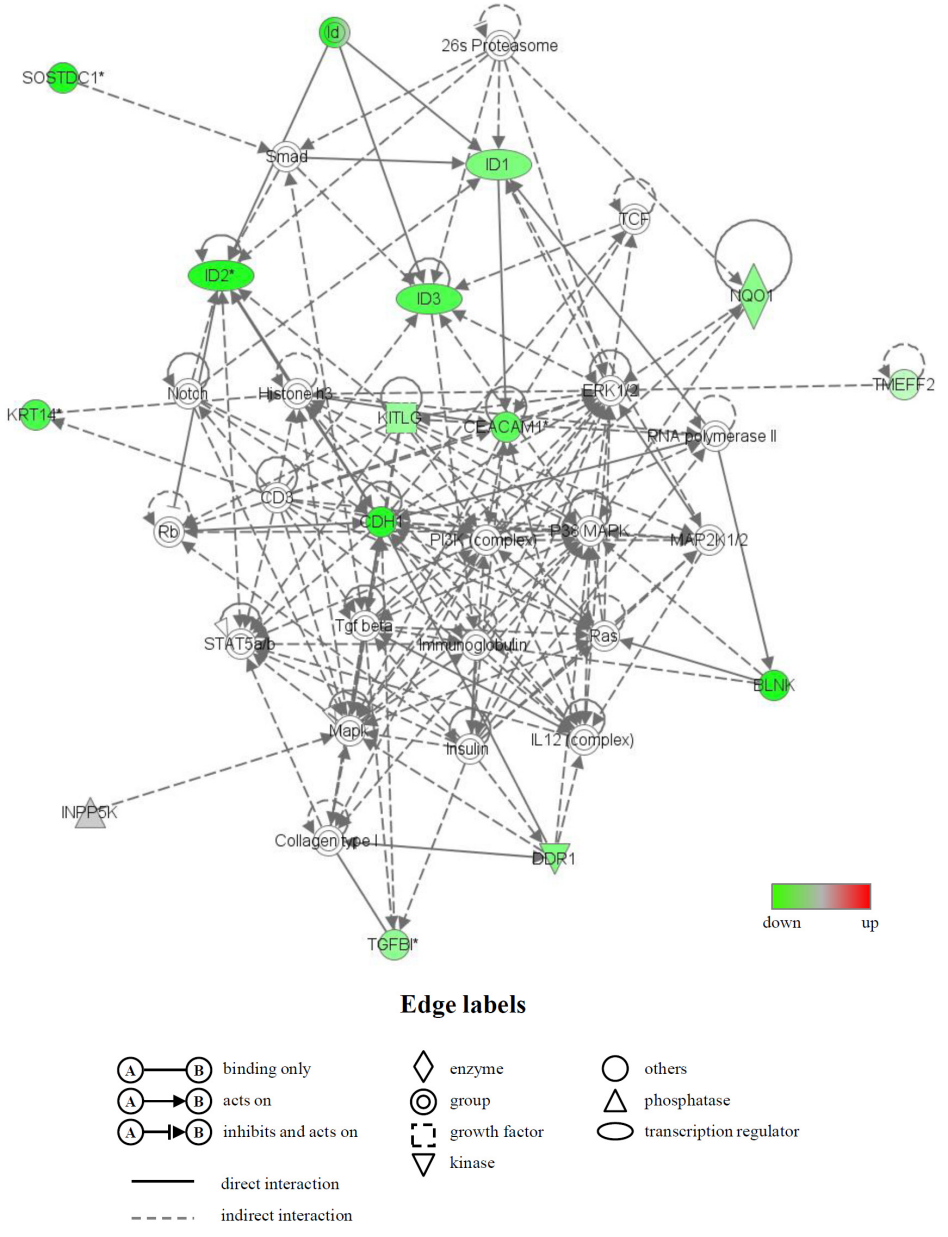
A gene network of genes that were downregulated in the LIPUS-treated cells. Downregulated genes were analyzed by Ingenuity^®^ Pathways Analysis tools. The gene network DOWN is shown. For an explanation of the symbols and letters, see Figure 4.

Moreover, the biologically relevant networks of the differentially expressed genes identified from the GeneChip^®^ analysis were depicted using the Ingenuity^®^ Pathways Knowledge Base. Two significant gene networks, U (up) and D (down), were obtained from the functionally annotated upregulated and downregulated genes, respectively (Figures 4 and 5). The gene network U contained CD200 antigen (*Cd200*), dentin matrix protein 1 (*Dmp1*), fibrillin 1 (*Fbn1*), insulin-like growth factor 2 (*Igf2*), lumican (*Lum*), matrix metallopeptidase 13 (*Mmp13*), osteomodulin (*Omd*) and thrombospondin 1 (*Thbs1*) and was associated with biological functions, such as cardiovascular disease, skeletal and muscular system development and function and organ morphology. In this network, six genes (*Dmp1*, *Fbn1*, *Igf2*, *Lum*, *Mmp13* and *Thbs1*), whose names are highlighted in blue, were mainly associated with bone morphology (functions annotation) in the category of biological functions (skeletal and muscular system development and function) (Figure 4). The gene network D contained inhibitor of DNA binding (Id) genes, *Id1*, *Id2* and *Id3*, and keratin 14 (*Krt14*) and was associated with biological functions, such as gene expression, cell cycle and connective tissue development and function (Figure 5).

### 2.5. Quantitative Analysis of Differentially Expressed Genes

**Figure 6:**
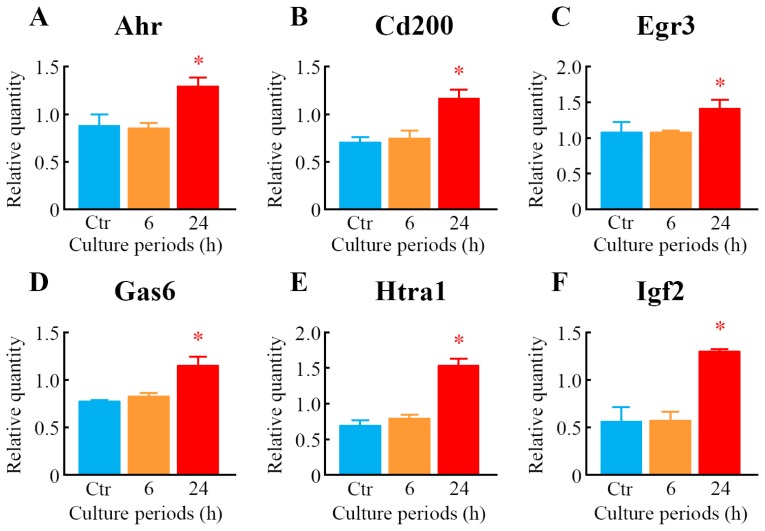

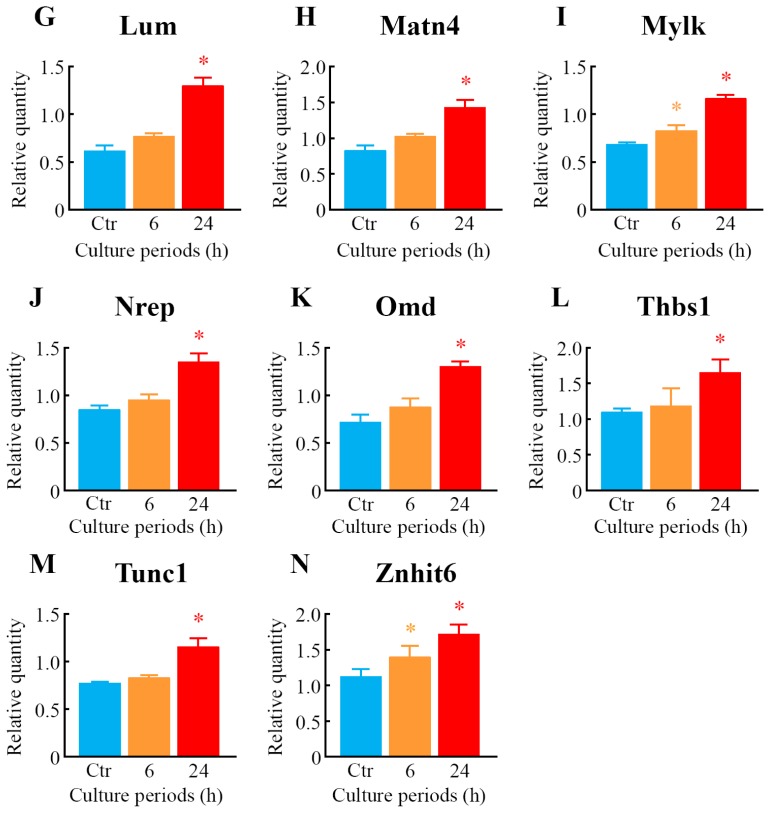
Verification of upregulated genes as judged by microarray analysis using real-time quantitative PCR. After treatment of the cells with LIPUS at 30 mW/cm^2^ for 20 min, the cells were cultured for 6- and 24-h at 37 °C. Real-time quantitative PCR was carried out with specific primers for *Ahr* (**A**); *Cd200* (**B**); *Egr3* (**C**); *Gas6* (**D**); *Htra1* (**E**); *Igf2* (**F**); *Lum* (**G**); *Matn4* (**H**); *Mylk* (**I**); *Nrep* (**J**); *Omd* (**K**); *Thbs1* (**L**); *Tunc1* (**M**) and *Znhit6* (**N**). Mock-treated cells served as a control (Ctr). Data indicate the means ± SD for four different experiments. * *p* < 0.05 *vs.* Ctr by Student’s *t*-test.

**Figure 7:**
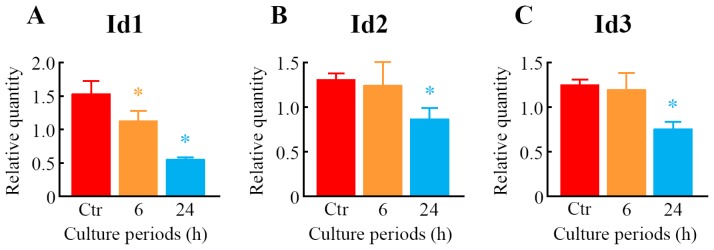
Verification of downregulated genes as judged by microarray analysis using real-time quantitative PCR. After treatment of the cells with LIPUS at 30 mW/cm^2^ for 20 min, the cells were cultured for 6- and 24-h at 37 °C. Real-time quantitative PCR was carried out with specific primers for *Id1* (**A**); *Id2* (**B**) and *Id3* (**C**). Mock-treated cells served as a control (Ctr). Data indicate the means ± SD for four different experiments. * *p* < 0.05 *vs.* Ctr by Student’s *t*-test.

To further explore the results of GeneChip^®^ analysis, a real-time quantitative polymerase chain reaction (PCR) assay was carried out. Seventeen genes were selected from among the genes that were upregulated or downregulated in response to LIPUS treatment. The expression levels of 14 genes, *i.e.*, aryl-hydrocarbon receptor (*Ahr*), *Cd200*, early growth response 3 (*Egr3*), growth arrest specific 6 (*Gas6*), HtrA serine peptidase 1 (*Htra1*), *Ifg2*, *Lum*, matrilin 4 (*Matn4*), myosin, light polypeptide kinase (*Mylk*), neuronal regeneration related protein (*Nrep*), *Omd*, *Thbs1*, troponin C, cardiac/slow skeletal (*Tnnc1*) and zinc finger, HIT type 6 (*Znhit6*), were significantly upregulated (Figure 6), whereas three genes, such as *Id1*, *Id2*, and *Id3*, were significantly downregulated in the cells treated with LIPUS (Figure 7). After 6-h LIPUS treatment, significant upregulations of *Mylk* and *Znhit6* and downregulation of *Id1* were detected (Figures 6 and 7). These data were similar to the results of microarray analysis.

## 3. Discussion

Within the fields of orthopedics, LIPUS has been used to promote bone fracture healing [3–6]. Although numerous studies have been conducted on this issue [7–38], the molecular mechanisms by which LIPUS affects bone cells have not been well understood. The use of *in vitro* cell culture systems has been of central importance for research at the cellular and molecular levels. The present study employed high-density oligonucleotide microarray analysis combined with computational gene expression analysis tools to demonstrate, for the first time, that many genes were responsive to LIPUS in MC3T3-E1 preosteoblast cells.

A number of previous reports [24–39] clearly indicated that LIPUS had positive effects on differentiation, gene expression, mineralization and proliferation under *in vitro* cell culture conditions, although the degree of these effects differed among studies. It is assumed that differences in the experimental conditions, such as the intensity of LIPUS, exposure period, cell type and culture conditions, contributed to discrepancies in the biological effects of LIPUS reported. Unsworth *et al.* [27] demonstrated that the ALP activity was significantly increased at days 6 to 10, but not at days 2 and 4 in MC3T3-E1 cells treated with once daily LIPUS (30 mW/cm^2^ for 20 min). Using rat osteoblastic ROS 17/2.8 cells, Takayama *et al.* [26] found that ALP activity was increased at day 7 of culture after transient LIPUS (30 mW/cm^2^ for 20 min) stimulation without affecting the cell proliferation. A single LIPUS (30 mW/cm^2^ for 20 min) treatment induces RANKL and chemokines via the angiotensin II type 1 receptor, while this stimulation does not affect the expressions of ALP and Bglap in MC3T3-E1 cells [25]. In mouse bone-marrow-derived stromal ST2 cells, once LIPUS (30 mW/cm^2^ for 20 min) exposure induced significant increases in the expressions of *Bglap*, as well as FBJ osteosarcoma oncogene (*Fos*), an immediate-early gene [32]. In this study, although neither cell growth nor ALP activity was influenced by a single exposure of MC3T3-E1 cells to LIPUS (30 mW/cm^2^ for 20 min), significant elevation of the mRNA level of *Bglap* was observed in the LIPUS-treated cells.

In the present study, many of the 38 and 37 genes that were upregulated or downregulated by 1.5-fold or more, respectively, were identified in the cells after LIPUS treatment. Seventeen genes were validated by real-time quantitative PCR assay, which was consistent with the microarray data. Of these 75 genes, *Mmp13* and *Dmp1* are indicated to be related to LIPUS [27,37,42,43]. The expression level of *Mmp13* was reported to be affected by LIPUS; LIPUS (30 mW/cm^2^ for 20 min per day) significantly increased the expression of *Mmp13* mRNA in MC3T3-E1 cell culture at day 10 [27] and in nucleus pulposus HNPSV-1 cell culture at day 3 [37], whereas LIPUS (7.5, 30 and 120 mW/cm^2^) significantly and dose-dependently inhibited *Mmp13* expression in isolated rat chondrocytes [42]. Recent findings demonstrated no statistically significant difference between the control and LIPUS (30 mW/cm^2^)-treated groups in the expression of *Dmp1* on a human tooth slice organ culture [43]. To the best of our knowledge, 73 genes out of 75 genes were not previously reported as related to the LIPUS response.

In addition, to study the molecular functions and gene networks, the microarray data were analyzed using Ingenuity*^®^* Pathway Analysis tools. Of particular interest in this study was the identification of gene network U, which contained many upregulated genes that were principally associated with bone morphology. Six of the genes in this network, *Dmp1* [44], *Fbn1* [45], *Igf2* [46], *Lum* [47], *Mmp13* [48] and *Thbs1* [49], have previously been shown to be involved in bone morphology. In addition, a number of genes in the gene network U, including *Cd200*, *Dmp1*, *Igf2* and *Nr4a1*, have been shown to be expressed in osteoblasts [50] and to participate in the development and/or differentiation of osteoblasts. For example, the Cd200-Cd200 receptor axis was shown to be a possible regulator of bone mass, via the formation of osteoclasts in this study using Cd200-knockout mice [51]. A significant increase in the expression of *Dmp1*, an intermediate and late marker of bone cell differentiation, was detected in fully differentiated osteoblasts [50], and the addition of Dmp1 protein to osteoblast cells markedly induced cell differentiation [52]. We also identified gene network D, which included many downregulated genes. Interestingly, this network contained the Id genes (other designation: inhibitor of differentiation), *Id1*, *Id2* and *Id3*, all of which belong to the helix-loop-helix (HLH) transcription factors and can form heterodimers among the basic HLH transcription factors [53]. All Id family members are reported to decrease the differentiation of bone cells, including osteoblasts [53,54]. The genes that were differentially expressed and/or belonged to gene networks are likely to be involved in the acceleration of fracture healing by LIPUS. However, further experiments will be needed to confirm this.

There are two research papers investigating the effects of LIPUS on gene expression in osteoblastic cells using global-scale microarray analysis. Leskinen *et al.* [28] indicated that 377 genes were regulated two-fold by a single LIPUS (1.035 MHz; mean acoustic peak pressure, 128–510 kPa; for 30 min) in human osteoblastic osteosarcoma MG-63 cells. They also suggested that LIPUS affects the genes involved with the cellular membrane and regulation of transcription, but not with osteoblast differentiation [28]. Lu *et al.* [29] identified 165 genes whose expressions were changed more than two-fold in human osteoblastic osteosarcoma SaOS-2 cells treated with a single LIPUS (30 mW/cm^2^ for 20 min). These genes belonged to more than ten protein families, including integrins and cytoskeleton genes, the transforming growth factor-beta family and the insulin-like growth factor family [29]. Moreover, Kobayashi *et al.* [37] previously showed using cDNA microarrays that LIPUS (30 mW/cm^2^ for 20 min) once daily for three days significantly induced 114 genes that were differentially expressed, and these genes included growth factor-related proteins, proteoglycans, collagens and matrix metallopeptidases. We identified 193 genes that were downregulated more than 1.5-fold and 201 genes that were upregulated more than 1.5-fold using a GeneChip microarray system in human lymphoma U937 cells treated with a single LIPUS (300 mW/cm^2^ for 1 min) [39]. Here, a total of 75 genes were differentially expressed in preosteoblast MC3T3-E1 cells at 24 h after LIPUS (30 mW/cm^2^ for 20 min) exposure. However, almost all of the genes that were differentially expressed and identified here were not reported in these four microarray studies [28,29,37,39]. As in the case of the biological effects of LIPUS, this discrepancy may have been due to the different experimental conditions, particularly with respect to the cell origin. Elucidation of the detailed mechanisms underlying the changes in gene expression responsive to LIPUS remains for further investigations.

## 4. Experimental Section

### 4.1. Cell Culture

The mouse preosteoblast cell line, MC3T3-E1, was provided by the: RIKEN BioResource Center (RIKEN BRC) through the National Bio-Resource Project of the Ministry of Education, Culture, Sports, Science and Technology of Japan (MEXT; Tsukuba, Japan). The cells were routinely cultured in minimum essential medium with alpha modification (MEMα; Wako Pure Chemical Industries, Ltd., Osaka, Japan) supplemented with 10% fetal bovine serum (FBS; Equitech-Bio, Inc., Kerrville, TX, USA) at 37 °C in humidified air with 5% CO_2_.

### 4.2. LIPUS Treatment

LIPUS treatment was applied by a sonic accelerated fracture-healing system (SAFHS) apparatus (SAFHS 4000J; Teijin Pharma, Ltd., Tokyo, Japan). This apparatus is the same as Exogen’s SAFHS apparatus (Exogen Inc., Piscataway, NJ, USA). The signal had a spatial average-temporal average (SATA) intensity of 30 mW/cm^2^, with a frequency of 1.5 MHz in a pulsed-wave mode (0.2-s burst sine waves repeated at 1.0 kHz).

**Figure 8:**
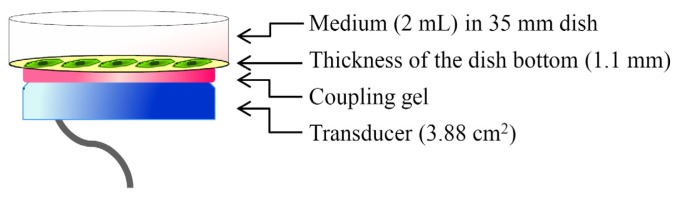
Illustration of the LIPUS apparatus.

The cells were cultivated in MEMα supplemented with 10% FBS, 0.3 mM L-ascorbic acid (Wako Pure Chemical Industries, Ltd., Osaka, Japan) and 10 mM β-glycerophosphate (Calbiochem, La Jolla, CA, USA) at 37 °C for 14 days. After trypsinization with 0.25% trypsin-0.91 mM EDTA solution, the cells (5 × 10^5^ cells) were seeded on a 35-mm plastic culture dish (ASAHI GLASS Co., Ltd., Tokyo, Japan) with 2 mL of culture medium and cultured at 37 °C for 24 h. It has been demonstrated that LIPUS is applied to cells from the upper [26,38] or lower side [25,27–29,31,33,35–37,39] of the culture dish. As demonstrated in Figure 8, LIPUS was transmitted through the bottom of the culture dish with standard US coupling gel (Teijin Pharma, Ltd., Tokyo, Japan). Attached cells in the dish were sonicated for 20 min at 37 °C in a CO_2_ incubator. After sonication, the cells were incubated at 37 °C in a CO_2_ incubator for the indicated period before the experiments.

### 4.3. Measurement of Cell Number

The cells were exposed to LIPUS followed by 0, 6, 24 and 48 h culture at 37 °C, were washed once with Ca^2+^/Mg^2+^-free phosphate buffered saline (PBS) and, then, treated with 0.25% trypsin-0.91 mM EDTA solution. The number of detached cells was counted using a Burker Turk hemocytometer.

### 4.4. Measurement of ALP Activity

The cells were exposed to LIPUS followed by 0, 6, 24 and 48 h culture at 37 °C, were washed once with Ca^2+^/Mg^2+^-free PBS and scraped using a plastic policeman. Cellular material was placed into 50 mM Tris-HCl buffer (pH 7.2) containing 0.01% Triton X-100 and homogenated by an ultrasonic disruptor (UD-200, TOMY SEIKO Co., Tokyo, Japan). The protein concentration was measured using a BCA Protein Assay Kit (Pierce; Rockford, IL, USA). ALP activity of the cell homogenate was measured by using a protocol supplied by Sigma (Sigma-Aldrich, Co., St. Louis, MO, USA).

### 4.5. RNA Isolation

The cells were exposed to LIPUS followed by 6 and 24 h culture at 37 °C. Total RNA was extracted from cells using an RNeasy Total RNA Extraction Kit (Qiagen K.K., Tokyo, Japan) along with on-column DNase I treatment (RNase-free DNase kit, Qiagen K.K., Tokyo, Japan). RNA quality was analyzed using a Bioanalyzer 2100 (Agilent Technologies, Inc., Santa Clara, CA, USA). RNA samples that had RNA integrity number (RIN) values above 9.5 were considered acceptable.

### 4.6. High-Density Oligonucleotide Microarray and Computational Gene Expression Analyses

Gene expression was analyzed using a GeneChip^®^ system with a Mouse Genome 430 2.0 array (Affymetrix, Inc., Santa Clara, CA, USA) spotted with 45,101 probe sets. Samples for array hybridization were prepared as described in the Affymetrix GeneChip^®^ Expression Technical Manual. In short, 500 ng of total RNA were used to synthesize cRNA with a GeneChip^®^ 3′ IVT Express Kit (Affymetrix, Inc., Santa Clara, CA, USA). After fragmentation, biotin-labeled cRNA was hybridized to the array at 45 °C for 16 h. The arrays were washed, stained with streptavidin-phycoerythrin and scanned using a probe array scanner. The obtained hybridization intensity data were further analyzed using GeneSpring^®^ GX (Agilent Technologies, Inc., Santa Clara, CA, USA) to extract the significant genes. To examine gene ontology, including biological processes, cellular components, molecular functions and gene networks, the obtained data were analyzed using Ingenuity^®^ Pathway Analysis tools (Ingenuity Systems, Inc., Mountain View, CA, USA), a web-delivered application that enables the identification, visualization and exploration of molecular interaction networks in gene expression data [39–41].

### 4.7. Real-Time Quantitative PCR Assay

Complementary DNA was produced from total RNA using an ExScript RT Reagent Kit (Takara Bio Inc., Shiga, Japan) with a random 6 mers and an oligo dT primer. Real-time quantitative PCR was performed on an Mx3005P real-time PCR system (Agilent Technologies, Inc., Santa Clara, CA, USA) using SYBR^®^ PreMix ExTaq (Takara Bio Inc., Shiga, Japan). The specific primer and probe sequences are listed in Table S3. The temperature cycling conditions for each primer consisted of 10 s at 95 °C followed by 40 cycles of 15 s at 95 °C and 40 s at 60 °C. The dissociation analysis was carried out over a range from 55 °C to 95 °C by monitoring SYBR green fluorescence, and PCR-specific products were determined as a single peak in the melting curves at more than 80 °C. In addition, the specificity of primers was confirmed as a single band with the correctly amplified fragment size through an agarose gel electrophoresis of the PCR products. Glyceraldehyde-3-phosphate dehydrogenase (*Gapdh*) was used as an internal control.

### 4.8. Statistical Analysis

Results were expressed as the means ± standard deviation (SD). Differences in the means were analyzed using the Student’s *t*-test, and *p* values < 0.05 were regarded as statistically significant.

## 5. Conclusions

In conclusion, we discovered a number of differentially expressed genes in MC3T3-E1 preosteoblast cells treated with LIPUS. These results should help to further clarify the molecular basis of the mechanisms of the LIPUS response in osteoblast cells.

## Supplementary Information



## Figures and Tables

**Table 1:** List of upregulated probe sets.

Probe set ID	Gene symbol	6 h		24 h		Gene title

		Mean	SD	Mean	SD	
1417399_at	*Gas6*	1.04	0.13	1.53	0.05 *	growth arrest specific 6
1454214_a_at	*Znhit6*	1.11	0.02 *	1.55	0.10 *	zinc finger, HIT type 6
1423854_a_at	*Rasl11b*	1.16	0.12	1.58	0.10 *	RAS-like, family 11, member B
1425505_at	*Mylk*	0.95	0.04 *	1.58	0.05 *	myosin, light polypeptide kinase
1455040_s_at	*Nhsl2*	0.90	0.21	1.59	0.13 *	NHS-like 2
1456295_at	*B230114P17Rik*	1.24	0.10	1.63	0.06 *	RIKEN cDNA B230114P17 gene
1417937_at	*Dact1*	1.01	0.08	1.67	0.11 *	dapper homolog 1, antagonist of beta-catenin (*Xenopus*)
1421811_at	*Thbs1*	1.11	0.12	1.68	0.26 *	thrombospondin 1
1426208_x_at	*Plagl1*	1.74	0.06	1.71	0.02 *	pleomorphic adenoma gene-like 1
1448171_at	*Siah2*	1.01	0.76	1.71	0.18 *	seven in absentia 2
1448788_at	*Cd200*	1.24	0.08	1.72	0.11 *	CD200 antigen
1433454_at	*Abtb2*	1.29	0.12	1.73	0.12 *	ankyrin repeat and BTB (POZ) domain containing 2
1441368_at	-	1.07	0.05	1.74	0.10 *	-
1442157_at	-	1.14	0.06 *	1.74	0.16 *	-
1416225_at	*Adh1*	1.20	0.20	1.75	0.25 *	alcohol dehydrogenase 1 (class I)
1416505_at	*Nr4a1*	1.12	0.07	1.76	0.03 *	nuclear receptor subfamily 4, group A, member 1
1418464_at	*Matn4*	1.08	0.05	1.76	0.19 *	matrilin 4
1422631_at	*Ahr*	0.98	0.04	1.76	0.09 *	aryl-hydrocarbon receptor
1423261_at	*1500015O10Rik*	1.00	0.15	1.78	0.27 *	RIKEN cDNA 1500015O10 gene
1423607_at	*Lum*	0.97	0.03	1.79	0.22 *	lumican
1450839_at	*D0H4S114*	1.07	0.06	1.80	0.17 *	DNA segment, human D4S114
1436329_at	*Egr3*	1.02	0.12	1.81	0.23 *	early growth response 3
1438870_at	*Fbn1*	1.11	0.11	1.82	0.30 *	fibrillin 1
1447927_at	*Gbp10*	1.12	0.38	1.82	0.13 *	guanylate-binding protein 10
1418252_at	*Padi2*	1.08	0.15	1.86	0.23 *	peptidyl arginine deiminase, type II
1418745_at	*Omd*	1.05	0.05	1.87	0.18 *	osteomodulin
1438251_x_at	*Htra1*	0.89	0.04	2.02	0.16 *	HtrA serine peptidase 1
1448152_at	*Igf2*	0.95	0.08	2.04	0.69	insulin-like growth factor 2
1416749_at	*Htra1*	1.00	0.14	2.06	0.06 *	HtrA serine peptidase 1
1420448_at	*Rhox2a*	1.20	0.41	2.08	0.66	reproductive homeobox 2A
1427149_at	*Plekha6*	0.87	0.33	2.10	0.77	pleckstrin homology domain containing, family A member 6
1460049_s_at	*1500015O10Rik*	1.05	0.10	2.12	0.17 *	RIKEN cDNA 1500015O10 gene
1427053_at	*Abi3bp*	1.16	0.23	2.19	0.84	ABI gene family, member 3 (NESH) binding protein
1416371_at	*Apod*	1.00	0.04	2.26	0.31 *	apolipoprotein D
1443746_x_at	*Dmp1*	1.10	0.16	2.29	0.19 *	dentin matrix protein 1
1426081_a_at	*Dio2*	1.03	0.17	2.38	0.55 *	deiodinase, iodothyronine, type II
1427054_s_at	*Abi3bp*	1.01	0.11	2.38	0.32 *	ABI gene family, member 3 (NESH) binding protein
1436996_x_at	*Lyz1*	0.86	0.51	2.41	0.76	lysozyme 1
1448929_at	*F13a1*	1.56	0.18	2.42	0.16 *	coagulation factor XIII, A1 subunit
1443745_s_at	*Dmp1*	1.11	0.08	2.44	0.35 *	dentin matrix protein 1
1426139_a_at	*Ccrl1*	1.09	0.04	2.47	0.94 *	chemokine (C-C motif) receptor-like 1
1417256_at	*Mmp13*	0.83	0.13	2.65	0.46 *	matrix metallopeptidase 13
1448326_a_at	*Crabp1*	1.18	0.08	2.68	0.50 *	cellular retinoic acid binding protein I
1418370_at	*Tnnc1*	0.92	0.09	2.82	0.08 *	troponin C, cardiac/slow skeletal
1418937_at	*Dio2*	0.97	0.04	3.05	0.36 *	deiodinase, iodothyronine, type II
1427338_at	*Crocc*	2.48	1.53	2.26	1.41	ciliary rootlet coiled-coil, rootletin

The data are expressed as the means ± standard deviation (SD) (*N* = 3).

* *p* < 0.05 (Student’s *t-*test).

**Table 2:** List of downregulated probe sets.

Probe set ID	Gene symbol	6 h		24 h		Gene title

		Mean	SD	Mean	SD	
1451780_at	*Blnk*	0.96	0.22	0.37	0.08 *	B-cell linker
1422537_a_at	*Id2*	1.05	0.29	0.40	0.04 *	inhibitor of DNA binding 2
1423935_x_at	*Krt14*	0.96	0.06	0.43	0.01 *	keratin 14
1425538_x_at	*Ceacam1*	1.05	0.39	0.45	0.07 *	carcinoembryonic antigen-related cell adhesion molecule 1
1425789_s_at	*Anxa8*	0.89	0.01 *	0.46	0.04 *	annexin A8
1448261_at	*Cdh1*	0.86	0.08	0.46	0.05 *	cadherin 1
1460347_at	*Krt14*	0.97	0.07	0.46	0.06 *	keratin 14
1435176_a_at	*Id2*	0.91	0.17	0.47	0.11 *	inhibitor of DNA binding 2
1460684_at	*Tm7sf2*	1.00	0.09	0.47	0.08 *	transmembrane 7 superfamily member 2
1416630_at	*Id3*	0.94	0.04	0.48	0.07 *	inhibitor of DNA binding 3
1418595_at	*Plin4*	1.12	0.51	0.50	0.16 *	perilipin 4
1460250_at	*Sostdc1*	1.02	0.05	0.50	0.08 *	sclerostin domain containing 1
1425895_a_at	*Id1*	0.98	0.13	0.51	0.16 *	inhibitor of DNA binding 1
1439382_x_at	*Ddr1*	0.96	0.20	0.51	0.21	discoidin domain receptor family, member 1
1428738_a_at	*D14Ertd449e*	1.00	0.28	0.52	0.09 *	DNA segment, Chr 14, ERATO Doi 449, expressed
1436520_at	*Ahnak2*	1.05	0.20	0.52	0.08 *	AHNAK nucleoprotein 2
1452656_at	*Zdhhc2*	0.98	0.24	0.53	0.12 *	zinc finger, DHHC domain containing 2
1424162_at	*Trim29*	0.80	0.34	0.54	0.16 *	tripartite motif-containing 29
1434046_at	*AA467197*	0.92	0.01 *	0.54	0.05 *	expressed sequence AA467197
1417732_at	*Anxa8*	0.90	0.08	0.55	0.06 *	annexin A8
1438152_at	-	1.35	0.57	0.55	0.13 *	-
1449060_at	*Kif2c*	0.82	0.08	0.55	0.10 *	kinesin family member 2C
1454702_at	*4930503L19Rik*	0.87	0.05 *	0.55	0.03 *	RIKEN cDNA 4930503L19 gene
1426152_a_at	*Kitl*	1.01	0.02	0.56	0.09 *	kit ligand
1458236_at	*-*	1.06	0.05	0.56	0.05 *	-
1415871_at	*Tgfbi*	0.90	0.06	0.57	0.04 *	transforming growth factor, beta induced
1427357_at	*Cda*	0.94	0.13	0.57	0.05 *	cytidine deaminase
1449340_at	*Sostdc1*	0.97	0.07	0.57	0.08 *	sclerostin domain containing 1
1419489_at	*Fam19a5*	0.96	0.06	0.58	0.03 *	family with sequence similarity 19, member A5
1422123_s_at	*Ceacam1*	0.87	0.26	0.58	0.06 *	carcinoembryonic antigen-related cell adhesion molecule 1
1456412_a_at	*Inpp5k*	0.71	0.21	0.58	0.04 *	inositol polyphosphate 5-phosphatase K
1432202_a_at	*Poc1a*	0.78	0.14	0.58	0.08 *	POC1 centriolar protein homolog A (Chlamydomonas)
1458385_at	*Hspa4l*	0.95	0.06	0.58	0.04 *	heat shock protein 4 like
1419073_at	*Tmeff2*	0.98	0.17	0.59	0.07 *	transmembrane protein with EGF-like and two follistatin-like domains 2
1440924_at	*Kif20b*	0.99	0.06	0.61	0.02 *	kinesin family member 20B
1417751_at	*Stk10*	0.95	0.16	0.62	0.03 *	serine/threonine kinase 10
1423627_at	*Nqo1*	1.02	0.02	0.62	0.05 *	NAD(P)H dehydrogenase, quinone 1
1452654_at	*Zdhhc2*	0.98	0.12	0.62	0.06 *	zinc finger, DHHC domain containing 2
1430193_at	*Casc5*	0.85	0.10	0.62	0.03 *	cancer susceptibility candidate 5
1430617_at	*Oip5*	0.92	0.12	0.62	0.04 *	Opa interacting protein 5
1417823_at	*Gcat*	0.86	0.14	0.63	0.03 *	glycine C-acetyltransferase (2-amino-3-ketobutyrate-coenzyme A ligase)
1423569_at	*Gatm*	1.08	0.04	0.64	0.02 *	glycine amidinotransferase (l-arginine: glycine amidinotransferase)
1456250_x_at	*Tgfbi*	0.94	0.03	0.64	0.02 *	transforming growth factor, beta induced
1435554_at	*Tmcc3*	0.93	0.08	0.64	0.02 *	transmembrane and coiled coil domains 3
1452968_at	*Cthrc1*	0.99	0.02	0.64	0.02 *	collagen triple helix repeat containing 1
1436654_at	*Gen1*	1.00	0.13	0.65	0.01 *	Gen homolog 1, endonuclease (Drosophila)
1456569_x_at	*Gsn*	0.58	0.08*	5.62	8.87	gelsolin
1436622_at	*Iqsec2*	0.62	0.01*	0.98	0.21	IQ motif and Sec7 domain 2

The data are expressed as the means ± standard deviation (SD) (*N* = 3).

* *p* < 0.05 (Student’s *t*-test).
