# Supplementary material for: Genes Responsive to Low-Intensity Pulsed Ultrasound in MC3T3-E1 Preosteoblast Cells
Source: Int J Mol Sci. 2013 Nov 18;14(11):22721–40. doi: 10.3390/ijms141122721 (PMC3856087; doi:10.3390/ijms141122721)
Supplement: Supplementary file 1 [file ijms-14-22721-s001.pdf]

Supplementary Information

Table S1. Functionally annotated genes and top five biological functions in upregulated probe sets.

Name	*p*-value	Genes (number of genes)
All functionally annotated genes		*ADH1C*, *AHR*, *APOD*, *C2orf40*, *CCRL1*, *CD200*, *CRABP1*, *DACT1*, *DIO2*, *DMP1*, *EGR3*, *F13A1*, *FBN1*, *GAS6*, *HTRA1*, *IGF2*, *LUM*, *Lyz1/Lyz2*, *MMP13*, *MYLK*, *NR4A1*, *NREP*, *OMD*, *PADI2*, *PLAGL1*, *PLEKHA6*, *SIAH2*, *THBS1*, *TNNC1 (29)*
Skeletal and muscular system development and function	5.26E-07-1.80E-02	*AHR*, *APOD*, *CD200*, *DACT1*, *DMP1*, *EGR3*, *FBN1*, *GAS6*, *IGF2*, *LUM*, *MMP13*, *MYLK*, *NR4A1*, *PLAGL1*, *THBS1*, *TNNC1 (16)*
Cellular movement	5.30E-06-1.80E-02	*AHR*, *CCRL1*, *CD200*, *DMP1*, *F13A1*, *FBN1*, *GAS6*, *HTRA1*, *IGF2*, *LUM*, *Lyz1/Lyz2*, *MMP13*, *MYLK*, *NR4A1*, *NREP*, *SIAH2*, *THBS1 (17)*
Connective tissue development and function	3.08E-05-1.80E-02	*AHR*, *DACT1*, *DMP1*, *FBN1*, *GAS6*, *IGF2*, *LUM*, *MMP13*, *NR4A1*, *PLAGL1*, *SIAH2*, *THBS1 (12)*
Embryonic development	3.08E-05-1.80E-02	*AHR*, *DACT1*, *DIO2*, *DMP1*, *EGR3*, *FBN1*, *GAS6*, *IGF2*, *LUM*, *MMP13*, *MYLK*, *NR4A1*, *PLAGL1*, *THBS1 (14)*
Organ development	3.08E-05-1.80E-02	*AHR*, *DACT1*, *DIO2*, *DMP1*, *EGR3*, *FBN1*, *GAS6*, *IGF2*, *LUM*, *MMP13*, *MYLK*, *PLAGL1*, *THBS1 (13)*

Table S2. Functionally annotated genes and top five biological functions in downregulated probe sets.

Name	*p*-value	Genes (number of genes)
All functionally annotated genes		*ANXA8L2*, *BLNK*, *C15orf48*, *CDA*, *CDH1*, *CEACAM1*, *CTHRC1*, *DDR1*, *GATM*, *GCAT*, *GPAA1*, *HSPA4L*, *ID1*, *ID2*, *ID3*, *INPP5K*, *KIF20B*, *KIF2C*, *KITLG*, *KRT14*, *NQO1*, *SOSTDC1*, *TGFBI*, *TMCC3*, *TMEFF2*, *TRIM29*, *ZDHHC2 (27)*
Gene expression	1.43E-08-8.01E-03	*ID1*, *ID2*, *ID3*, *KITLG (4)*
Cell cycle	7.95E-07-1.99E-02	*BLNK*, *CDH1*, *CEACAM1*, *ID1*, *ID2*, *ID3*, *KIF20B*, *KIF2C*, *KITLG*, *KRT14 (10)*
Connective tissue development and function	7.95E-07-1.74E-02	*CDH1*, *CEACAM1*, *ID1*, *ID2*, *ID3*, *KITLG*, *NQO1 (7)*

Table S2. *Cont.*

Name	*p*-value	Genes (number of genes)
Cellular development	1.19E-06-2.20E-02	*BLNK*, *CDH1*, *CEACAM1*, *CTHRC1*, *DDR1*, *GCAT*, *ID1*, *ID2*, *ID3*, *INPP5K*, *KITLG*, *KRT14*, *NQO1*, *SOSTDC1*, *TGFBI*, *TMEFF2 (16)*
Cellular growth and proliferation	1.19E-06-2.20E-02	*BLNK*, *CDA*, *CDH1*, *CEACAM1*, *CTHRC1*, *DDR1*, *GCAT*, *ID1*, *ID2*, *ID3*, *INPP5K*, *KIF20B*, *KIF2C*, *KITLG*, *KRT14*, *NQO1*, *TGFBI*, *TMEFF2 (18)*

Table S3. Nucleotide sequences of primers and a probe for target genes.

Genes	Orientation	Nucleotide sequence (5' to 3')	GenBank accession No.
Ahr	Sense<br>Antisense	gtcacagcagatgccttggt<br>ccttgtgcagagtctgggtt	NM_013464
Bglap	Sense<br>Antisense	ctctgacctcacagatgcca<br>tttgtaggcggtcttcaagc	NM_007541
Cd200	Sense<br>Antisense	aaaggcgctgcacacaactg<br>gttttctgggctcacggctttc	NM_010818
Egr3	Sense<br>Antisense	atgggctccattccggaaca<br>aatcccgggtggatctgctt	NM_018781
GAPDH	Sense<br>Antisense	tcggtgtgaacggatttggc<br>gtgccgttgaatttgccgtg	NM_008084
Gas6	Sense<br>Antisense	tcctggcagttgaggatg<br>gccatccacttctagggt	NM_019521
Htra1	Sense<br>Antisense	tgacagagtcccacgatcgaca<br>ccacagactgtccgttgatgct	NM_019564
Id1	Sense<br>Antisense	gaacgtcctgctctacgaca<br>ccgacttcagactccgagtt	NM_010495
Id2	Sense<br>Antisense	gactcgcatcccactatcgtca<br>caggatgctgatgtccgtgttc	NM_010496
Id3	Sense<br>Antisense	cctcttggacgacatgaaccac<br>gtggcaaaagctcctcttgtcc	NM_008321
Igf2	Sense<br>Antisense	gggtggtaacacgatcagacgacttccccagatacc	NM_010514
Lum	Sense<br>Antisense	cacagctacccaactgccatgt<br>cactgcaggtctgtgacgttct	NM_008524
Matn4	Sense<br>Antisense	gccaaagaggaaggcatcgtca<br>ccggagaataggacacgtgcaa	NM_013592
Mylk	Sense<br>Antisense	ccatccgtgatctggaagtcgt<br>cagttgccgtcttcgtcgtagt	NM_139300
Nrep	Sense<br>Antisense	gtcagccaagaaccgtttg<br>actggtagctggagaggtga	NM_053078
Omd	Sense<br>Antisense	gtgagcagaggagtactaacgg<br>cctgactgtcatggtcgtct	NM_012050

Table S3. *Cont.*

Genes	Orientation	Nucleotide sequence (5' to 3')	GenBank accession No.
Spp1	Sense<br>Antisense<br>Probe	ggacctcacctctcacatgaaga<br>agacttggttcatccagctgact<br>FAM™-cagcttctgagcatgccctctgatca-TAMRA™	NM_009263
Thbs1	Sense<br>Antisense	acaggtgtgcaaaccgcgaaac<br>tgatgccattgcctgcatagcc	NM_011580
Tnnc1	Sense<br>Antisense	gttcggtgcatgaaggacgaca<br>tggcctgcagcatcatcttcag	NM_009393
Znhit6	Sense<br>Antisense	agtcacagactggggtccaa<br>cctctcaacaccacgtgcaa	NM_001081094

Figure S1. Venn diagram of probe sets that were differentially expressed. Cells were exposed to LIPUS (30 mW/cm2, for 20 min), followed by culturing at 37 °C for six (**A** and **B**) and 12 h (**C** and **D**). Gene expression analysis of the probe sets that were upregulated (A and C) and downregulated (B and D) by a factor of 1.5 or greater was conducted using GeneSpring® software. The diagram shows the number of specifically and commonly expressed probe sets affected by LIPUS. The experiments were repeated three times.

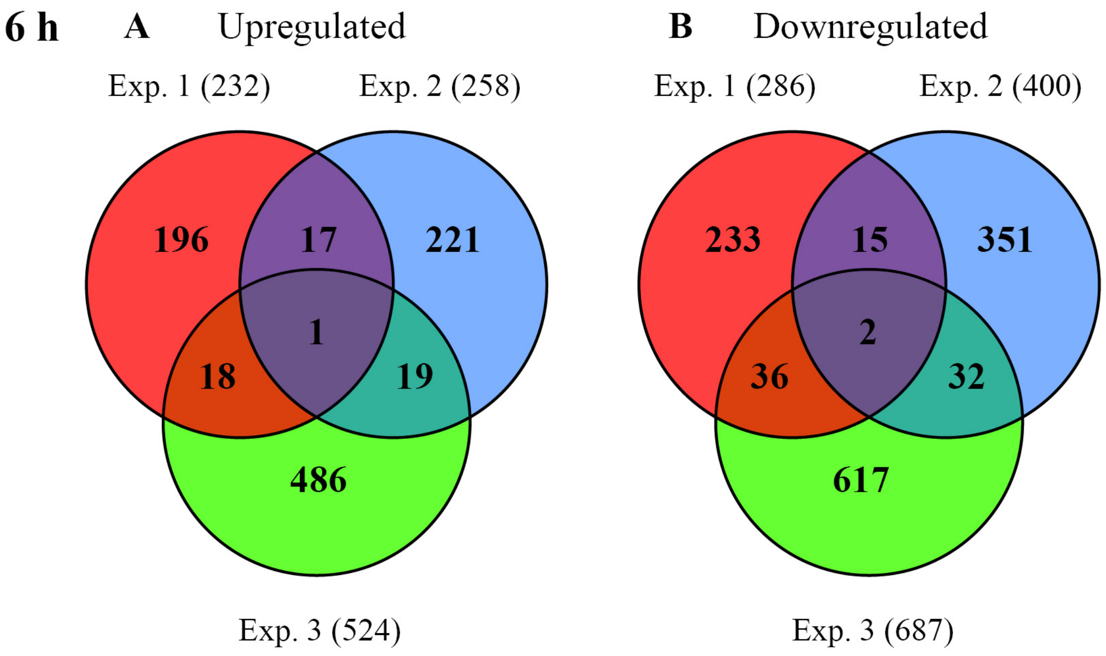

Figure S1. *Cont.*

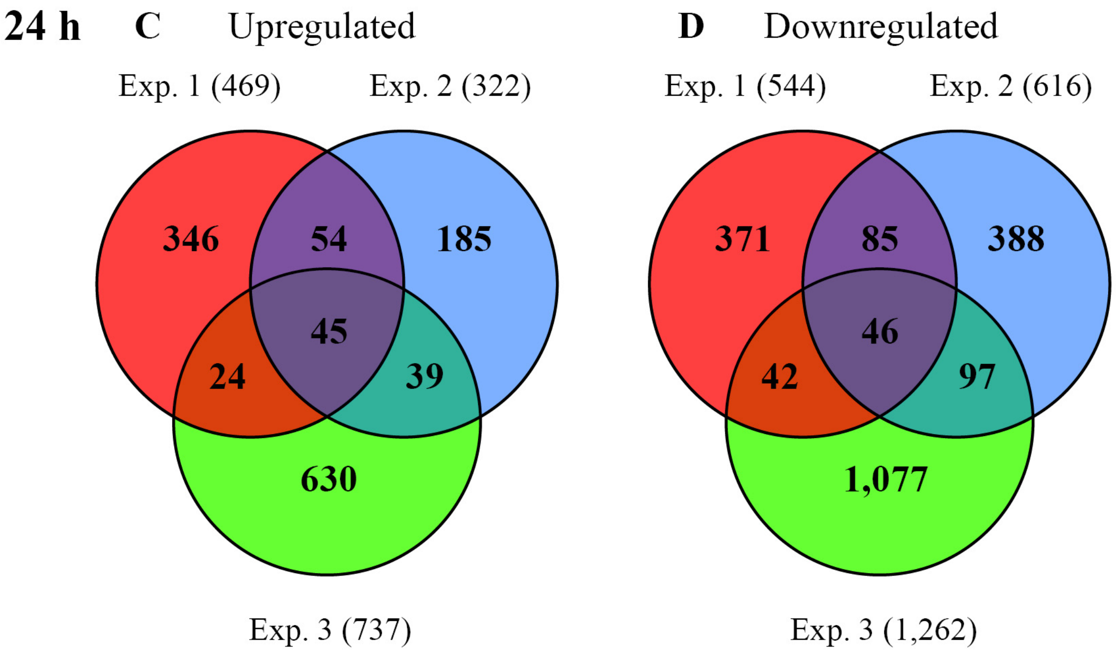

© 2013 by the authors; licensee MDPI, Basel, Switzerland. This article is an open access article distributed under the terms and conditions of the Creative Commons Attribution license (http://creativecommons.org/licenses/by/3.0/).
